# *In vivo* efficacy and molecular docking of designed peptide that exhibits potent antipneumococcal activity and synergises in combination with penicillin

**DOI:** 10.1038/srep11886

**Published:** 2015-07-09

**Authors:** Cheng-Foh Le, Mohd Yasim Mohd Yusof, Mahmood Ameen Abdulla Hassan, Vannajan Sanghiran Lee, Diyana Mohd Isa, Shamala Devi Sekaran

**Affiliations:** 1 Department of Medical Microbiology, Faculty of Medicine, University of Malaya, Kuala Lumpur, Malaysia; 2 Department of Biomedical Science, Faculty of Medicine, University of Malaya, Kuala Lumpur, Malaysia; 3 Department of Chemistry, Faculty of Science, University of Malaya, Kuala Lumpur, Malaysia

## Abstract

We have previously designed a series of antimicrobial peptides (AMPs) and in the current study, the *in vivo* therapeutic efficacy and toxicity were investigated. Among all the peptides, DM3 conferred protection to a substantial proportion of the lethally infected mice caused by a strain of penicillin-resistant *Streptococcus pneumoniae.* Synergism was reported and therapeutic efficacy was significantly enhanced when DM3 was formulated in combination with penicillin (PEN). No toxicity was observed in mice receiving these treatments. The *in silico* molecular docking study results showed that, DM3 has a strong affinity towards three protein targets; autolysin and pneumococcal surface protein A (pspA). Thus AMPs could serve as supporting therapeutics in combination with conventional antibiotics to enhance treatment outcome.

For decades, *Streptococcus pneumoniae* has been recognized as one of the major human bacterial pathogen responsible for serious invasive pneumococcal infections such as meningitis, sepsis, and pneumonia^1^,^2^,^3^,^4^,^5^. According to World Health Organization WHO, 0.7–1 million deaths out of the 1.6 million total deaths due to pneumococcal diseases were in children aged less than five years^6^. Children from developing countries are the most heavily impacted group by pneumococcal diseases^7^. *S. pneumoniae* represents the leading etiological agent causing death due to acute bacterial respiratory infection and nonepidemic meningitis in children in developing countries^8^,^9^. Although different classes of antibiotics are currently available, ß-lactam antibiotics including penicillin, cephalosporins, and carbapenems remain as the preferred choice of antibiotics against pneumococcal infections^10^. Of concern, the emergence of antibiotic-resistant *S. pneumoniae* has increased the disease burden of pneumococcal infections^11^. Increasing antibiotic resistance especially with *S. pneumoniae* has been a major concern as ineffective therapy would delay disease resolution and dramatically increase the risks of complication and death^12^. Treatment failure is associated with increased healthcare financial burden and the risks of morbidity and mortality^13^,^14^,^15^,^16^.

The discovery of antimicrobial peptides (AMPs) and its potential application as novel therapeutic agents have been expanding rapidly in recent years^17^,^18^. AMPs exhibit broad spectrum antimicrobial activity against a wide diversity of gram-positive and gram-negative bacterial species, fungi, as well as eukaryotic parasites and enveloped viruses^19^,^20^,^21^,^22^. From isolation of AMPs from natural sources and determination of their biological activity, researchers are investing intense efforts into developing synthetic analogues of AMPs with enhanced antimicrobial activity while reducing cell toxicity effects to be developed as clinically usable therapeutic agents. A surfactant containing the 18 residues N-terminal fragment of Prophenins has been suggested as a potential local treatment agent for pulmonary infections^23^. In addition, findings on peptides producing synergism with standard drugs have been reported. For example, magainin 2 and PGLa are able to interact cooperatively in a synergistic manner against bacteria, artificial membranes, and also tumor cells^24^. Magainin 2 is a slow pore former but the pores formed remain stable and long-lived. LL-37 exhibits broad spectrum antibacterial activity^25^ and the antimicrobial activity can be further synergized in the presence of defensins^26^.

We have previously designed a series of five synthetic hybrid peptides (DM1, DM2, DM3, DM4, and DM5) which showed strong antipneumococcal activity irrespective of the strains’ penicillin susceptibility. In this study, the therapeutic efficacy of the peptides will be investigated using two highly lethal mouse infection models (pneumonia and systemic) infected by a penicillin-resistant pneumococcal strain. The two infection models were chosen to reflect the clinical manifestations of lethal pneumococcal infections in human. Treatment doses of each peptide were chosen based on the results from the non-toxic dose determined in *in vivo* toxicity testing. We found DM3 to show moderate level of therapeutic efficacy and in combination with penicillin was dramatically superior than the standalone treatment.

## Results

### *In vivo* toxicity of the hybrids was minimal

*In vivo* toxicity of the five DMs was assessed following the three dose regimens given to the mice at 2 hrs, 12 hrs and 24 hrs. For mice treated via subcutaneous (SC) route at the highest dose of 100 mg/kg, no physical/behavioral abnormality was observed and no death occurred up to day 7 post treatment. No significant histological abnormality was observed in the spleen, kidney, liver, lung, and brain of the mice as compared to the untreated control. Only mice treated with DM1 (high urea level) and DM2 (low plasma protein and creatinine levels) were reported with minor blood parameter changes (Table 1). Similarly, mice treated with DM1 (low thrombocytes count) and DM2 (low thrombocytes count and low alkaline phosphatase level) via intranasal (IN) route at the highest dose of 20 mg/kg were seen with minor blood parameters changes. In addition, mice treated with DM5 were found to have lower creatinine levels (p = 0.028, 22.5 ± 11.73 μmol/l) than the untreated control (45.5 ± 11.24 μmol/l).

For treatment via intraperitoneal (IP) route, lower graded doses were selected for DM1 (5 mg/kg), DM2 (60 mg/kg), DM3 (40 mg/kg). DM4 (5 mg/kg), and DM5 (20 mg/kg) as higher doses caused death and/or high physical/behavioral stress in the mice. The MCV level was lower than the untreated control for DM3 and DM5-treated mice (Table 1). For DM4-treated mice, the AST level was reduced significantly. No histopathological abnormality was observed as compared to the untreated control. Among the four DM3-PEN formulations, only mice receiving DM3_20_—PEN_20_ was observed with lower urea levels. No significant histological abnormality was noted.

### DM3 showed therapeutic efficacy in pneumococcal systemic infection model

**Figure 1:**
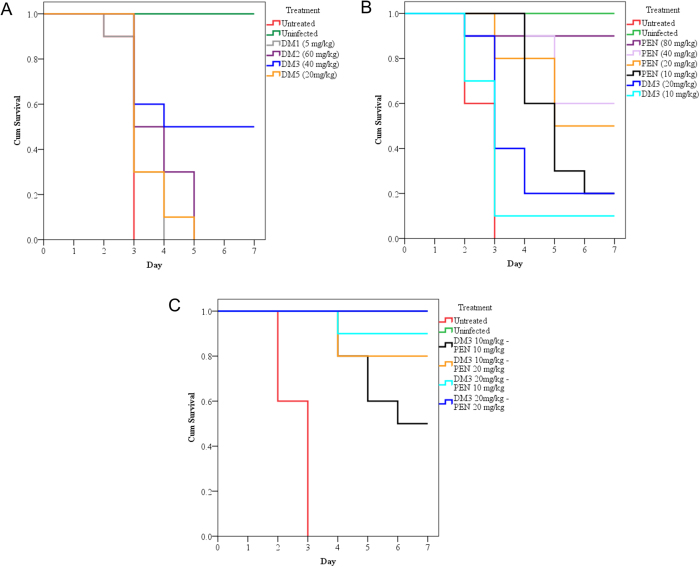
Survival function analysis of mice with lethal pneumococcal systemic infection treated with the hybrids, PEN, and in combination. Treatments were administered for three doses regimen (2 hrs, 12 hrs, 24 hrs) via IP route postinfection. Statistical analysis was performed for each treated group versus untreated group using Kaplan-Meier with log-rank test (Mantel-Cox). (**A**) DM3 treatment at 40 mg/kg conferred significant survival function (p = 0.004) with 50% survival rate up to day 7 postinfection. Mice treated with DM2 (p = 0.009) and DM5 (p = 0.045) showed statistically significant enhanced survival although no survival was noted up to day 7 postinfection. (**B**) PEN treatment at 10 mg/kg, 20 mg/kg, 40 mg/kg, and 80 mg/kg showed highly significant survival function (p < 0.001) with 20%, 50%, 60%, 90% survival rates up to day 7 postinfection. While for lower graded DM3 doses of 10 mg/kg and 20 mg/kg, there were 10% (p = 0.424) and 20% (p = 0.019) survival rates, respectively. (**C**) The combination treatments showed significant (P < 0.001) survival function and 50%, 80%, 90%, and 100% survival rates were achieved with DM3_10_—PEN_10_, DM3_10_—PEN_20_, DM3_20_—PEN_10_, and DM3_20_—PEN_20_, respectively.

The therapeutic efficacy of the peptides was tested in two infection models. The inoculum was optimized to produce lethal infection and caused death within two to four days post infection. Typical physical and behavioural presentations associated with severe pneumococcal infection were observed and confirmed by detection of pneumococci from the organ homogenates. The treatment regimens for each DM followed the highest deliverable doses based on the *in vivo* toxicity assessment. In pneumococcal systemic infection model, preliminary screening using groups of three mice found that mice treated with DM1, DM2, DM3, and DM5 at the respective doses conferred enhanced survival and were selected for testing using groups of 10 mice. Results showed that DM3 at 40 mg/kg protected 50% of the lethally infected mice from mortality for up to day 7 post infection with statistically significant survival function (p = 0.004) (Fig. 1A). Infected mice treated with DM1, DM2, and DM5 were all dead between days 4 to day 5 post infection. Nevertheless, survival analysis showed that treatment using DM2 (p = 0.009) and DM5 (p = 0.045) significantly enhanced survival of the systemically infected mice as compared to the untreated control.

Mice receiving PEN treatments via IP route at 10 mg/kg, 20 mg/kg, 40 mg/kg, and 80 mg/kg were determined to have 20%, 50%, 60%, and 90% survival rates up to day 7 post infection and were statistically significant (p < 0.001) (Fig. 1B). Mice receiving the lower graded doses of DM3 at 10 mg/kg and 20 mg/kg had 10% (p = 0.424) and 20% (p = 0.019) survival rates at day 7 post infection. The 20 mg/kg dose treatment was statistically significant (p = 0.019) as compared to the untreated group. Mice surviving up to day 7 were humanely sacrificed and no growth of *S. pneumoniae* was obtained in blood and in five organs. No survival was recorded in mice treated via SC or IN routes for all DMs tested. In the pneumococcal pneumonia model, no survival was observed for all treatment regimens attempted for each of the DMs.

### DM3 showed therapeutic synergism in combination with PEN

DM3 and PEN displayed potent therapeutic efficacy in a synergistic fashion. Treatment with low doses of standalone DM3 and PEN conferred only minimal survivability as combinations of DM3_10_—PEN_10_ and DM3_10_—PEN_20_ protected 50% and 80% of the mice lethally infected in the pneumococcal systemic infection model (Fig. 1C). Remarkably, maximal protection (90% and 100%) were noted in mice receiving the DM3_20_—PEN_10_, and DM3_20_—PEN_20_ treatments. Survival function of these four groups were statistically significant (p < 0.001). Moreover, the survival rates were higher than the sum of the survival rates for the respective peptide/antibiotic in its standalone form by 20%, 20%, 50%, and 30%, for DM3_10_—PEN_10_, DM3_10_—PEN_20_, DM3_20_—PEN_10_, and DM3_20_—PEN_20_ groups respectively. In addition, mice surviving the lethal infection remained physically active with no sign of physical or behavioral abnormality.

### Pathological sequelae reduced substantially in mice receiving DM3 and DM3+PEN treatments

**Figure 2:**
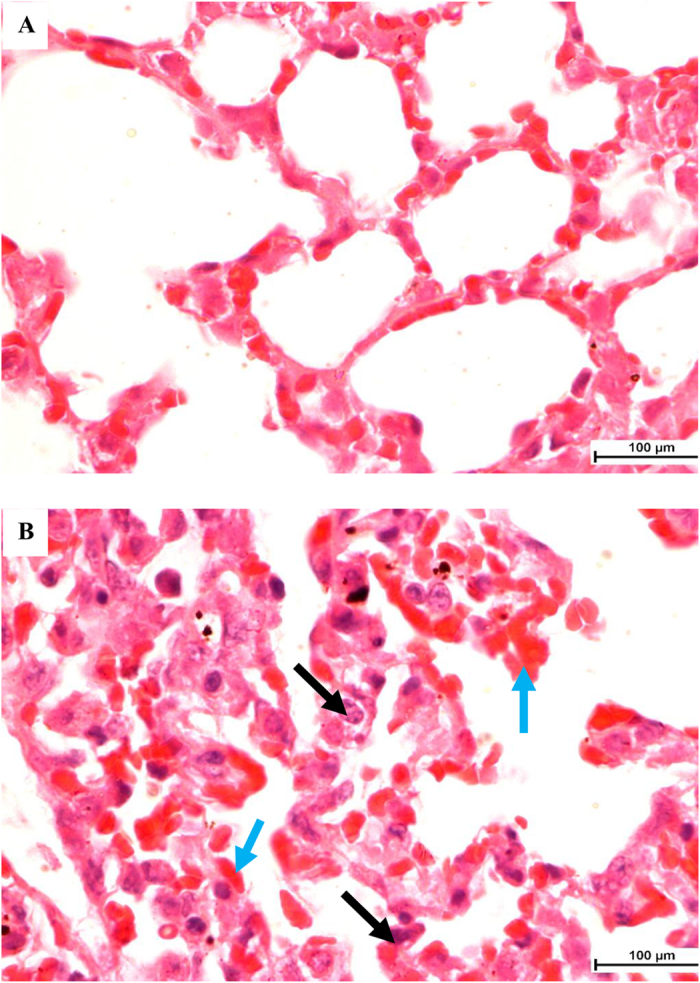
Histology of lung tissues from uninfected and lethally infected mice by *S. pneumoniae*. As compared to the (**A**) uninfected control, the lung from (**B**) infected control displayed significant changes with thickening of alveolar wall and excessive alveolar exudates filled with inflammatory cells mainly the neutrophils (black arrow). The infiltrations of erythrocytes into the alveolar wall and alveolar spaces strongly indicate pulmonary hemorrhage. Magnification at 1000X, H & E staining. Bar indicates 100 μm.

**Figure 3:**
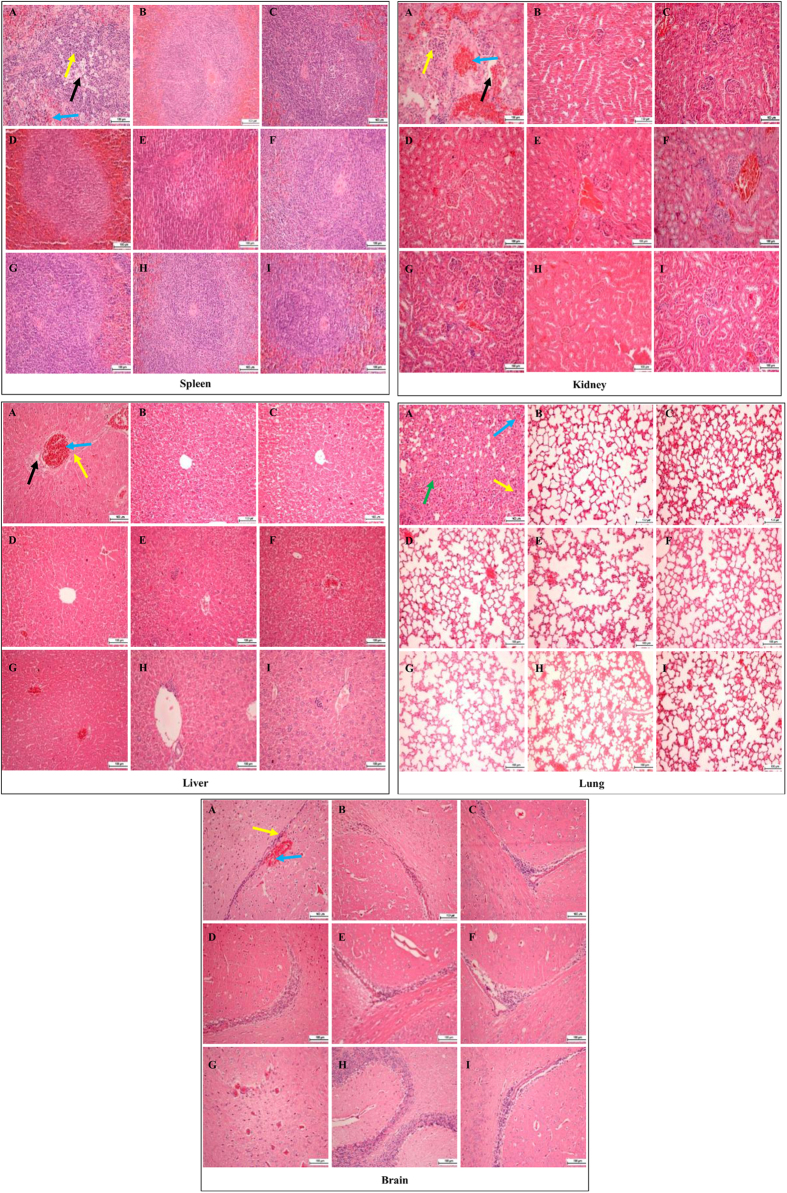
Histology of five major organs of mice lethally infected by *S. pneumoniae* receiving treatments. Images showing tissues from (**A**) infected control and (**B**) uninfected control (normal) mice as compared to mice treated with (**C**) DM3 at 10 mg/kg, (**D**) DM3 at 20 mg/kg, (**E**) DM3 at 40 mg/kg, (**F**) DM310—PEN10, (**G**) DM310—PEN20, (**H**) DM320—PEN10, and (**I**) DM320—PEN20. Organs from the infected control mice showed multiple lesions (yellow arrow), infiltration of erythrocytes and vascular congestion (blue arrow), and damage of tissue structure (black arrow). Heavy alveolar congestion in the lung can be observed (green arrow). These histological changes in the treated mice were significantly minimal as compared to the inected control mice. Magnification at 200X, H & E staining. Bar indicates 100 μm.

Histological examinations of the systemically infected mice with and without treatment are shown in Fig. 2. Overall, the infected organs showed severe inflammation and tissue damages. Lungs represent the most severely affected organ with significant post infection changes. As compared to the uninfected normal mice (Fig. 2A), several pathological changes were noted in the lung of the infected control mice including high vascular congestion, highly thickened alveolar wall with foci consolidation due to excessive capillary congestion and edema, and alveolar exudates filled with inflammatory cells mainly neutrophils and macrophages (Fig. 2B, black arrow). Heavy infiltrations of erythrocytes into the alveolar spaces and pulmonary tissues strongly indicate pulmonary hemorrhage (Fig. 2B, blue arrow). This is of significant contrast to the uninfected mice displaying the highly aerated alveolar spaces with thin layer of alveolar wall in the normal lung. The highly congested lung can be seen with little alveolar spaces (Figs 2 and 3—Lung, A). The exudative process is typical of bacterial lung infection and in the current study, it was *S. pneumoniae* as confirmed by plating the homogenates of the organs on blood agar. Severe tissue damage was also seen with the spleen especially at the white pulp area (Fig. 3—spleen, A, black arrow).

In addition, the infected control mice displayed acute glomerulonephritis as observed with severe inflammation causing damage to large areas with loss of glomerular structures (Fig. 3—kidney, A, black arrow). The inflammatory cells and erythrocytes can be seen infiltrating the affected area (Fig. 3—kidney, A, yellow and blue arrows) as compared to the normal kidney with intact glomerular structures (Fig. 3—kidney, B). The formation of large lesions can be clearly observed in the kidney of infected control mice (Fig. 3—kidney, A, yellow arrow). Epithelial cells surrounding the lumen were damaged and are surrounded by inflammatory cells. In the brain, the presence of erythrocytes were detectable (Fig. 3—brain, A, blue arrow) although the extent of inflammation was less significant as compared to the other organs.

For several treatments including DM3 at 10 mg/kg, 20 mg/kg, and 40 mg/kg as well as the combination treatments of DM3_10_—PEN_10_, DM3_10_—PEN_20_, DM3_20_—PEN_10_, and DM3_20_—PEN_20_, it was observed that the quantity and severity of lesions were significantly lower than the infected control mice. For instance, all the lungs of the treated mice showed only low level vascular congestion and slight thickening of the alveolar wall even though these conditions were still detectable in the mice (Fig. 3—lung, C to I). The inflammation and damages were minimal as compared to the infected lung which showed high level congestion. In the liver, the epithelial cells and hepatocytes surrounding the vascular lumen can be seen damaged with large lesions (Fig. 3—liver, A) as compared to the other groups of mice which had only small lesions with few inflammatory cells. Furthermore, the treated mice had significantly lower lesions quantitatively in the liver as well. Similarly, the glomerular filtration unit and the proximal/distal convoluted tubules in the kidney of treated mice appeared normal with no major inflammation/lesions. Also no hemorrhage or only minimal in the treated mice as compared to the infected control mice was observed.

### Possible interactions with protein targets from *in silico* molecular docking study

**Figure 4:**
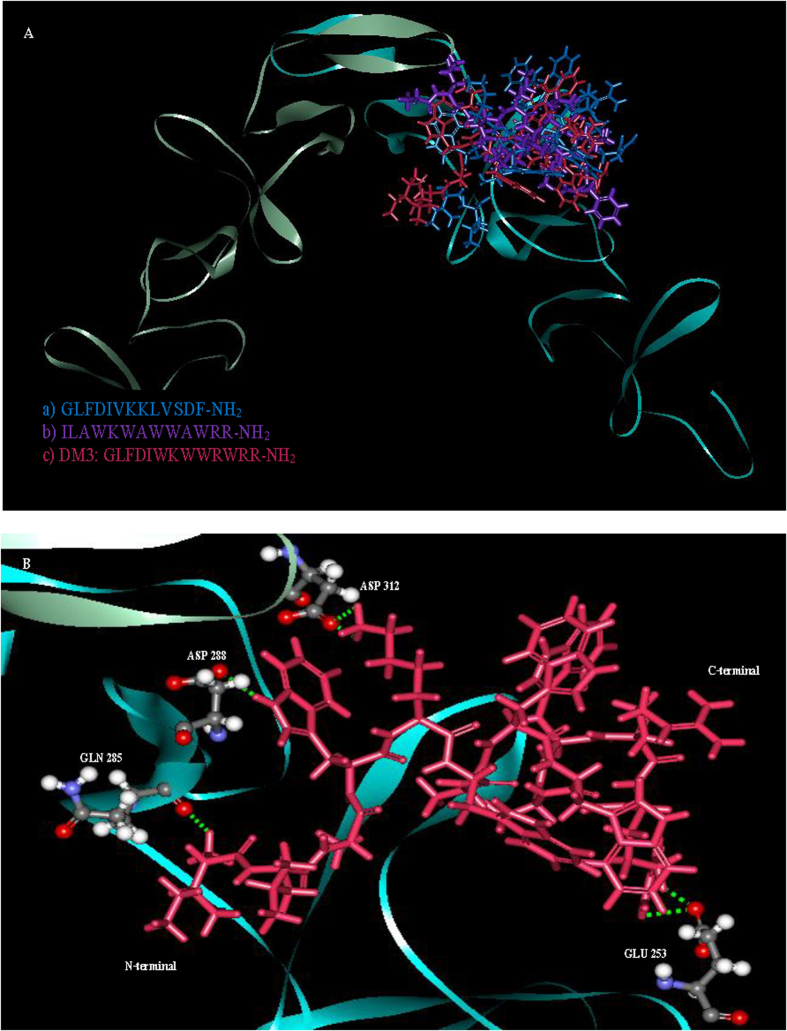
The interaction between autolysin and three peptides. **A** The superimposed autolysin with 1HR1 (blue), 1VM4 (purple) and DM3 (pink). **B** Hydrogen bondings interaction of DM3 were observed at DM3:LYS5:HZ1-A:ASP312:OD1, DM3:LYS5:HZ2-A:ASP312:OD1, DM3:ILE1:HT1-B:GLN285:O, DM3:TRP4:HE1-B:ASP288:OD1, DM3:ARG13:HH12-B:GLU253:OE1 and DM3:ARG13:HH22-B:GLU253:OE1. Amino acids with strong interaction (< -20 kcal/mo) with DM3 were labeled.

**Figure 5:**
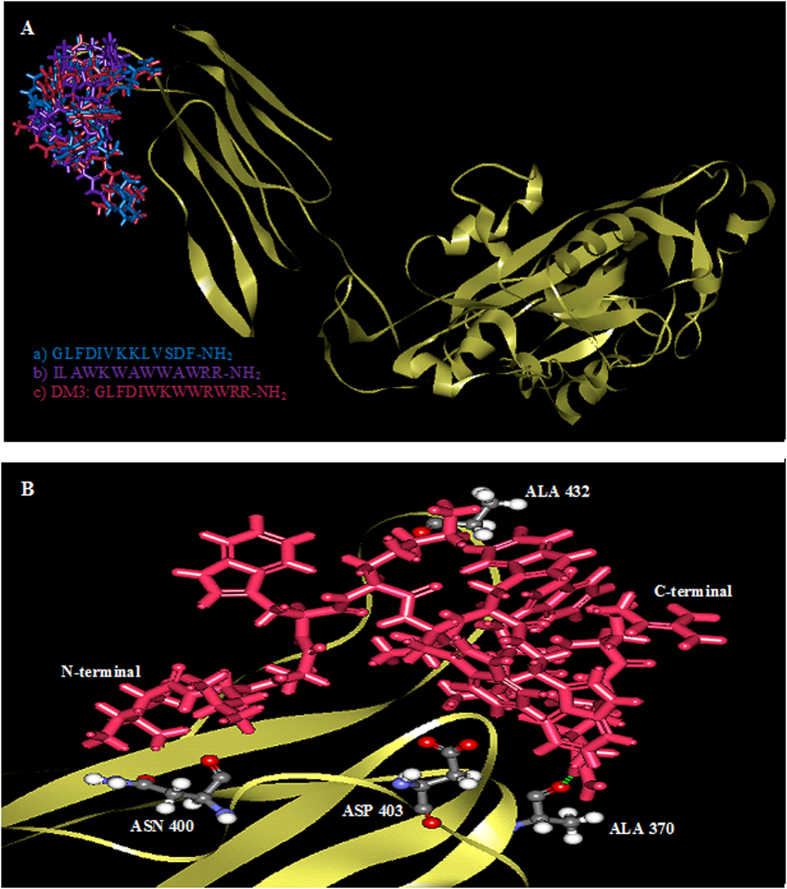
The interaction between pneumolysin and three peptides. (**A)** Superimposed of peptide a) (blue), peptide b) (purple) and c) DM3 (pink) and (**B**) binding amino acids and hydrogen bonding interaction in green found only at DM3:ARG13-HH22-A:ALA370:O. Amino acids with strong interaction (<−20 kcal/mo) with DM3 were labeled.

**Figure 6:**
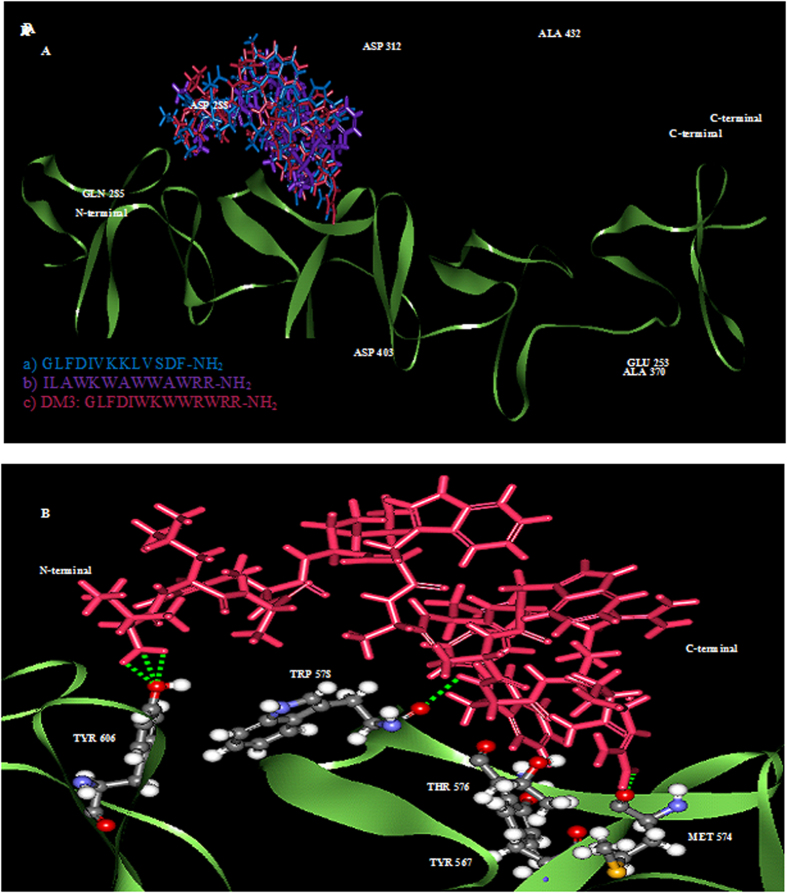
The interactions between pspA and three peptides. (**A)** Superimposed of peptide a) (blue), peptide b) (purple) and c) DM3 (pink) and (**B**) binding amino acids and interactions of hydrogen bonding in green observed at DM3:ILE1:HT1-A:TYR606:OH, DM3:ILE1:HT2-A:TYR606:OH, DM3:ILE1:HT3-A:TYR606:OH, DM3:TRP11:HN-A:TRP578:O and DM3:ARG13:HH12-A:MET574:O. Amino acids with strong interaction (<−20 kcal/mo) with DM3 were labeled.

Molecular docking allowed us to identify the interaction of peptides to the binding sites. The x-ray structure of homodimer in chain A and B was used for autolysin while pneumolysin and pneumococcal surface protein A (pspA) were homology modeled with SWISS-MODEL where the peptides was based on NMR solution structure data of indolicidin peptide derivative. The structures of three protein receptors were evaluated to ensure the model quality (data not shown). The docking result with rigid docking indicated that the peptides are active with the negative binding affinity range −7.8—(−2.7), −10.2—(−7.0), −7.8—(−2.7) in Table 2 for all three targets, autolysin, pneumolysin, and pspA. A low (negative) energy indicates a stable system and thus a likely binding interaction. The minimized lowest docking energy complexes of peptides with CHARMm force field against autolysin, pneumolysin, and pspA were visualized in discovery studio in Figs 4, 5, 6, respectively. In the representation, peptides a) GLFDIVKKLVSDF-NH_2_ b) ILAWKWAWWAWRR-NH_2_ c) DM3: GLFDIWKWWRWRR-NH_2_ c) in blue, purple, and pink, respectively, were superimposed to compare their interactions. A close view of interactions has been depicted; whereas green dotted lines represented the hydrogen bonds. The details of van der Waal (VDW), electrostatic, binding interaction (BE) with amino acids in 3 Å vicinity of the peptides, and total interaction energy (IE) value were tabulated in Table 3. For autolysin, DM3 has the strongest binding interactions −653 kcal/mol (Table 3) which is relatively lower than antibacterial and antitumor peptide, which have the same N-terminal GLFDI (about 53 kcal/mol and indolicidin peptide derivative about 44 kcal/mol). Greater numbers of interactions of amino acids in chain B of autolysin have been observed with strong binding interaction (<−10 kcal/mol) such as ASP312 of chain A and ASN252, GLU253, TRP261, VAL261, GLN285, SER286, ASP288 and TYR293. The contribution of the interaction mainly comes from the electrostatic interaction rather than van der Waals. Several hydrogen bonding interactions in green of DM3 have been found in Fig. 4 at DM3:LYS5:HZ1-A:ASP312:OD1, DM3:LYS5:HZ2-A:ASP312:OD1, DM3:ILE1:HT1-B:GLN285:O, DM3:TRP4:HE1-B:ASP288:OD1, DM3:ARG13:HH12-B:GLU253:OE1 and DM3:ARG13:HH22-B:GLU253:OE1F to interaction with both end chains of the peptide. For pneumolysin and pspA, indolicidin peptide derivative bounded better than DM3 or the antibacterial or the antitumor peptide. Only one hydrogen bonding interaction at the ARG13 of DM3 between DM3:ARG13:HH22 and A:ALA370:O (Fig. 5) was found and implied a weak interaction of pneumolysin with the N-terminal of the peptide. The strong contributions (<−10 kcal/mol in Table 4) are from the interaction with ALA370, TYR371, TYR376, ASN400, ASP403, CYS428, ALA432, TRP435, TRP436 of pneumolysin. Several hydrogen bond interactions with N-terminal of DM3 were illustrated in Fig. 6 at DM3:ILE1:HT1-A:TYR606:OH, DM3:ILE1:HT2-A:TYR606:OH, DM3:ILE1:HT3-A:TYR606:OH, DM3:TRP11:HN-A:TRP578:O and DM3:ARG13:HH12-A:MET574:O and the strong interactions with TYR567, ASN569, MET574, THR576, TRP578, LYS580, VAL581, TRP585, THR604, TYR606, LEU632 are listed (Table 5).

## Discussion

The increasingly prevalent finding of multidrug and high-level antibiotic-resistance pneumococci has prompted the development of improved antimicrobial agents. Based on the potent antipneumococcal activity of our designed hybrid peptides, determining the *in vivo* therapeutic efficacy is highly anticipated. When tested in the mouse pneumococcal systemic infection model, DM3, in its standalone form, showed significant therapeutic potential curing 50% of the lethally infected mice as compared to the untreated vehicle without treatment intervention. Notably, combination formulation of DM3 with PEN enhanced treatment outcome via therapeutic synergism even at low doses. This is particularly evident as 90% to complete survivability was observed with two of the dose formulations. This is in sharp contrast to the standalone treatment with either compound .by 20%–50%. However, the actual synergistic effect of DM3_20_—PEN_20_ (30%) could have been underestimated as the maximum efficacy (100%) has been achieved. Due to the higher PEN dose used in DM3_20_—PEN_20_ as compared to DM3_20_—PEN_10_, it is not inappropriate to extrapolate a >50% difference in synergism and standalone treatment. Besides, DM2 and DM5 did delay deaths and produced significant survival function though not preventing death in this model.

The lower dose of both compounds in the formulations also implies lower risk of associated toxicity than a single high dose. For instance, DM3_10_—PEN_20_ and DM3_20_—PEN_10_ were as effective as a single high dose of 80 mg/kg PEN. Using both agents at 20 mg/kg combination was better than this high PEN dose. The combination treatments as well as the standalone DM3 were nontoxic to the mice. In particular, no nephrotoxicity and hepatotoxicity was detected. In general, there was minimal toxicity associated with the peptides at the MNTD doses chosen as noted from the blood and serum biochemical parameters of the respective groups. The causes of the changes are not fully understood and will need further detailed studies on the pharmacodynamics/pharmacokinetics of the peptides in an animal model.

As compared to PEN, it was found that the dose of DM3 used has greatest impact on the therapeutic effect of the combination formulations. Increasing the dose of standalone PEN by 10 mg/kg (20 mg/kg) would enhance the protection by 30%, giving the expected survival rate of 80% in DM3_10_—PEN_20_ group over the expected survival rate of 50% in DM3_10_—PEN_10_ group and this was observed in the *in vivo* therapeutic synergism experiments. Similarly, increasing the dose of standalone DM3 by 10 mg/kg from 10 mg/kg (10%) to 20 mg/kg (20%) would enhance the protection by 10%, giving the expected survival rate of 60% in DM3_20_—PEN_10_ over DM3_10_—PEN_10_ (50%). Interestingly, the DM3_20_—PEN_10_ group recorded 90% survival rate which is 30% higher than the expected value. In other word, changes in the dose of DM3 will have greater effect than PEN on the resulting therapeutic efficacy of the DM3-PEN formulation. However, the importance of PEN should not be neglected as both components are equally critical in the formulation.

Pneumococcus is able to spread and establish infection rapidly following initial invasive inoculation. This had been demonstrated in both infection models in the current study. Severe organ tissues inflammation and damages caused by *S. pneumoniae* in the lethally infected mice have been clearly demonstrated based on the histological findings. Interestingly, such pathological changes were minimal and dramatically less extensive in mice treated with DM3 and the four combination therapies with PEN. In addition, these mice have no apparent signs of physical or behavioral abnormality. Although minor histological changes were still observable in these mice, this shows that the pathological sequelae caused by the highly virulent *S. pneumoniae* infection were almost unavoidable once the bacteria spread to the organs. However, current treatments were able to significantly clear the invaded pneumococci from the tissues and reduce these damages to the minimal level.

Although DM3 showed promising therapeutic potential, we noted some limitations in its treatment effect. Firstly, the peptide did not show therapeutic activity in the pneumonia model where inoculation was done directly into the thoracic cavity to infect the lungs while treatment was administered at distant sites. This indicates that DM3 as well as other DMs might not have been circularized efficiently to the site of infection to clear the pneumococci. It is also possible that the bioavailability of peptides have been reduced by inactivation by blood or cellular components such as enzymatic digestion by peptidases. However, DM3 was effective in the systemic infection model. This could be due to both inoculation and treatment being carried out inside the same compartment and DM3 exhibited sufficiently high killing activity to induce local elimination of pneumococcal cells infecting the organs and limiting bacterial spread. To circumvent this problem, conjugating the peptide with a component that would enhance its transportation and enzymatic resistance would be desirable.

The protein-peptide interaction plays a significant role in structural based drug designing. Molecular docking study was used to clarify the binding mode of the peptides with three protein receptors; autolysin, pneumolysin, and PspA which are virulence factors. Taken together; our docking results show negative binding energy indicating the favorable binding of peptides with all three receptors. Detailed amino acids involved in binding of the minimized lowest docking complexes indicate that DM3 shows the best binding and correlated with our experimental results possibly due to the inhibition of autolysin receptor. However, although DM3 can bind with pneumolysin and pspA, we note that the indolicidin peptide derivative has better binding with these two receptors. The DM3 peptide also formed several hydrogen bonds at both ends with several protein residues and had a greater number of the strong interactions with autolysin in chain B, while amino acids at chain A stabilized the peptide-autolysin complex, mainly from the strong electrostatic and hydrogen bonding interactions.

In conclusion, the potential application of AMPs as standalone or combination therapeutic agents to support the course of conventional antibiotics treatment is encouraging despite seeing several shortcomings of the current design. The DMs can be redesigned further to improve its therapeutic efficacy with reduced toxicity.

## Methods

All peptides for *in vivo* testing were synthesized to >95% purity by Genscript (USA) using 9-fluorenylmethoxycarbonyl for solid phase peptide synthesis. Quality analyses of peptides were validated using high performance liquid chromatography and mass spectrometry.

### Bacterial culture and assay medium

A PRSP strain (PEN MIC = 2 μg/ml) maintained in the Department of Medical Microbiology, University of Malaya was obtained. The strain was previously isolated from a clinical sample and stored at the University of Malaya Medical Centre. Sheep blood agar was used for the cultivation of the pneumococci. The strain was stored in multiple vials in Brain Heart Infusion Broth supplemented with 10% (v/v) glycerol at −80 ^°^C to avoid repeated freeze-thawed cycles on the cells. All freeze-stocked strains were passaged twice prior to experimentation.

### *In vivo* model

#### Animal maintenance and handling

Male ICR mice (4 weeks old) were used throughout the *in vivo* studies. The mice were acclimatized for seven days prior to any experimentation. Mouse feeding pellet and water were provided *ad libitum*. Procedures involving SC and IN routes which requires anesthesia were given the standard dose of ketamine (Narketan®-10, 100 mg/kg) and xylazine (ilium xylazil-20, 10 mg/kg) combination via IP injection. All injections were performed using Myjector 1 ml U100 29G insulin syringe. All animal handling and experimental procedures were approved by the Faculty of Medicine Animal Care and Use Committee of University of Malaya, ethics number MP/05/05/2010/LCF(R) and all animal experiments were carried out in accordance with approved guidelines and regulations.

### *In vivo* toxicity assessment

*In vivo* toxicity was performed to determine the possible side effects associated with the administration of the DMs in mice. The mice were randomized and three to four mice per group were administered with the respective peptides for the three dose regimen at 2 hrs, 12 hrs, and 24 hrs via SC, IN, and IP routes. The peptides were initially given at high doses (100 mg/kg via IP and SC routes at 200 μl, 20 mg/kg via IN route at 200 μl) and gradually reduced to lower doses if death or high degree physical/behavioral stress were observed in the mice within seven days. Doses administered via IN route were limited by the low volume (20 μl) deliverable through the nasal cavity of mouse and thus the highest dose given was 20 mg/kg. The untreated control group was administered with sterile distilled water only. At the seventh day, all mice were sacrificed and blood was collected via cardiac puncture using 25G syringe attached to 1 ml needle. Whole blood for complete haematogram was collected in 500 μl in a dipotassium EDTA microtainer tube. Blood collected in another 1.5 ml tube was centrifuged at 8,000 rpm for 5 mins and the serum was aliquoted to a fresh 1.5 ml tube for serum biochemistry analysis. The whole blood parameters analyzed were red blood cells (RBC), white blood cells (WBC), B neutrophil, S neutrophil, lymphocytes, monocytes, eosinophil, basophil, and thrombocytes, hemoglobin (Hb) concentration, packed cell volume (pCV), mean corpuscular volume (MCV), mean corpuscular haemoglobin concentration (MCHC), and plasma protein concentration. Serum biochemistry analysis included the concentrations of alanine transaminase (ALT), ALP, aspartate aminotransferase (AST), creatinine, urea, lactate dehydrogenase (LDH), and direct and total bilirubin. Major organs (brain, lung, liver, kidney, spleen) were harvested and fixed in 10% (v/v) buffered formalin for at least seven days. Histological tissues were processed with hematoxylin and eosin stains at the histopathology laboratory at a Veterinary Laboratory Service Unit.

### *In vivo* therapeutic efficacy assessment

#### Lethal murine pneumococcal infection models

Each selected peptide was tested in two lethal pneumococcal infection models: systemic infection and pneumonia challenged by a highly virulent PRSP strain selected. The strain was subcultured twice on sheep blood agar at 37 ^°^C under 5% CO_2_ for 18–24 hrs. The bacteria was suspended and serially diluted in BHIB on ice and immediately inoculated into the mice at the desired routes. The inoculum used to induce lethal systemic infection was 1.5 × 102 CFU/mouse (100 μl) via IP injection. To induce pneumonia in mice, the inoculum used was 5 × 103 CFU/mouse (50 μl) via intrathoracic route. Both models produced 100% mortality between day 2 to day 4 post infection. Uninfected control group (medium only) with equal number of mice as the test groups were included in each trial.

All mice were randomized before receiving the first treatment dose and divided into the respective groups. The selected dose and regimens of treatment of the respective peptides to be used were first screened using groups of three mice. Selected peptides were then tested in a larger group setting of 10 mice. Graded doses of PEN were also tested to determine the therapeutic efficacy of this conventional antibiotic. Uninfected controls (medium only) and untreated controls (inoculums only) with equal number of mice as the test groups were included in each trial and treated with sterile distilled water only. Survival of mice were recorded for seven days or until dead/moribund, whichever was earlier. Blood and homogenates of the five major organs (spleen, liver, lung, kidney, brain) of the mice that survived were plated on blood agar to detect the presence of pneumococci.

### *In vivo* therapeutic synergism with PEN

After determining the *in vivo* therapeutic efficacy of peptides in standalone form, the protective effect of the DMs in combination with PEN was also investigated. The therapeutic efficacy (n = 10) of both DM4 and PEN against the mouse infection model was each tested at low doses of 10 mg/kg and 20 mg/kg following the same testing protocol. Survival of mice were recorded for seven days or until dead/moribund, whichever was earlier. Blood and homogenates of the five major organs (spleen, liver, lung, kidney, brain) of the mice that survived were plated on blood agar to detect the presence of pneumococci.

### *In silico* molecular docking study

Molecular docking techniques were used to simulate the designed hybrid peptides and receptors targeting the potential virulence factors of *S. pneumoniae* interaction. Three receptors which are autolysin, pneumolysin and pspA were used as target and docked with designed peptides. The high resolution of crystal structure of choline-binding domain of major pneumococcal autolysin (PDB ID: 1GVM) was obtained from the RCSB protein data bank (http://www.pdb.org). The waters and ligands were removed from the original crystal structure. Then, the initial structures were modified according to the CHARMm force field with partial charge Momany-Rone^27^ and minimizations of the structures were performed with RMS gradient tolerance of 0.1000 kcal/(mol x Angstrom) satisfied. Pneumolysin was homology modelled that was deposited in the SWISS-MODEL repository (UniProt: Q04IN8)^28^. Automated comparative modeling of three-dimensional (3D) protein structures for pspA (ALA453-VAL653) was built using SWISS-MODEL server (http://swissmodel.expasy.org)^29^. All model structures were minimized with the same protocol as for autolysin. The overall quality of the minimized model was evaluated to ensure the model quality by utilizing PROCHECK^30^ for evaluation of Ramachandran plot quality, PROSA^31^ for interaction energy testing and VERIFY3D^32^ for assessing the compatibility of each amino acid residue. Three peptides; a) antibacterial and antitumor peptide designed based on the N-terminal membrane anchor of E. coli enzyme IIA (Glucose), GLFDIVKKLVSDF-NH_2_ (PDB ID = 1VM4), b) indolicidin peptide derivative (ILAWKWAWWAWRR-NH_2_) with improved activity against gram-positive bacteria. (PDB ID = 1HR1), and c) the most active hybrid peptide with further mutations, DM3 (GLFDIWKWWRWRR-NH_2_) were investigated to clarify their interaction with autolysin, pneumolysin and pspA.

Docking of peptides into the targets was performed using AUTODOCK VINA^33^ with rigid docking and the low interaction complex structures were further minimized. The binding site for autolysin is at Chain B:LYS258-ALA277, for pneumolysin at ARG426-ARG437, for pspA at GLY577-LEU588 where the binding site of pneumolysin and pspA had been predicted from prosite (http://prosite.expasy.org/). Detailed interaction energy was investigated by calculating binding energies using the protocol from Discovery Studio (Accelry Inc, 2.5.5). This allowed us to estimate the interaction energy between the target protein and designed peptides.

### Statistical analysis

Statistical analysis was performed using SPSS 16. Results were expressed as mean ± SD. Statistical difference in whole blood haematogram and serum biochemistry parameters between the treated versus the untreated control groups were conducted using One-way ANOVA with *post-hoc* Dunnett-t test. For *in vivo* therapeutic efficacy and synergism testing, survival analysis of each treated versus untreated control groups were performed using Kaplan-Meier analysis with log-rank test (Mantel-Cox). Survival plots were generated from the SPSS based on Kaplan-Meier analysis.

## Additional Information

**How to cite this article**: Le, C.-F. *et al.* *In vivo* efficacy and molecular docking of designed peptide that exhibits potent antipneumococcal activity and synergises in combination with penicillin. *Sci. Rep.* **5**, 11886; doi: 10.1038/srep11886 (2015).

## Figures and Tables

**Table 1:** Whole blood haematogram and serum biochemistry for *in vivo* toxicity determination in mice treated with the individual peptides or DM3—PEN combination at the respective doses via different routes of administration.

**Route**				**Parameter**	**Mean ± SD**		
	**Treatment**^a^	**Dose (mg/kg)**			**Untreated**	**Treated**	**P value**
Subcutaneous	DM1	100	—	Urea	7.27 ± 0.93	9.45 ± 1.25	0.033
	DM2	100	—	Plasma protein	60.33 ± 3.51	53.67 ± 1.53	0.027
		100	—	Creatinine	40.67 ± 0.58	32.67 ± 0.58	0.004
Intranasal	DM1	20	—	Thrombocytes	1464.25 ± 209.39	1006.00 ± 197.73	0.012
	DM2	20	—	Thrombocytes	1464.25 ± 209.39	729.50 ± 253.50	<0.001
			—	ALP	136.25 ± 10.34	220.00 ± 16.00	0.026
	DM5	20	—	Creatinine	45.50 ± 11.24	22.50 ± 11.73	0.028
Intraperitoneal	DM3	40	—	MCV	57.75 ± 0.96	52.25 ± 2.63	0.002
	DM4	5	—	AST	98.05 ± 34.38	50.88 ± 10.05	0.007
	DM5	20	—	MCV	57.75 ± 0.96	53.00 ± 2.83	0.022
	DM3—PEN	20	20	Urea	8.83 ± 0.98	5.95 ± 1.08	0.001

^a^Given for 3 doses (2 hrs, 12 hrs, and 24 hrs).

Parameters with value <0.1 was defined as 0.1 to allow statistical analysis. Statistical analysis between treatment groups and untreated control was performed using One-way ANOVA with *post hoc* Dunnett-t test. Only statistically significant different groups (p ≤ 0.05) among all routes and peptides tested were summarized. Abbreviations: Mean corpuscular volume, MCV (fl); Thrombocytes (x10^9^/l); Plasma protein (g/l); Alkaline phosphatase, ALP (U/l); Aspartate aminotransferase, AST (U/l); Creatinine (μmol/l); Urea (mmol/l).

**Table 2:** Molecular docking results with Autodock Vina.

**Receptor**	**Peptides**	**Binding Affinity (kcal/mol)**
Autolysin	GLFDIVKKLVSDF-NH_2_	−7.8–(−5.3)
	ILAWKWAWWAWRR-NH_2_	−6.0–(−3.4)
	DM3: GLFDIWKWWRWRR- NH_2_	−5.6–(−2.7)
Pneumolysin	GLFDIVKKLVSDF-NH_2_	−8.4–(−7.0)
	ILAWKWAWWAWRR-NH_2_	−10.2–(−7.6)
	DM3: GLFDIWKWWRWRR- NH_2_	−10.2–(−7.7)
PspA	GLFDIVKKLVSDF-NH_2_	−7.8–(−5.3)
	ILAWKWAWWAWRR-NH_2_	−6.0–(−3.4)
	DM3: GLFDIWKWWRWRR- NH_2_	−5.6–(−2.7)

**Table 3:** Contribution of the interactions energy in kcal/mol of the autolysin binding residues in the 3 Å from peptides.

**Residue**	**Interaction Energy (IE)**	**VDW**	**Electrostatic**	**Residue**	**Interaction Energy (IE)**	**VDW**	**Electrostatic**	**Residue**	**Interaction Energy (IE)**	**VDW**	**Electrostatic**
GLFDIVKKLVSDF-NH_2_				ILAWKWAWWAWRR-NH_2_				DM3: GLFDIWKWWRWRR- NH_2_			
A_ASP312	−61.07	−2.34	−58.72	A_LEU314	−1.95	−1.02	−0.93	A_ASP312	−128.63	0.50	−129.13
A_LEU314	−6.74	−4.00	−2.75	B_THR259	−16.45	−2.05	−14.41	A_LEU314	0.59	−1.67	2.25
B_TYR250	−10.00	−1.79	−8.20	B_GLY260	−2.22	−1.88	−0.34	B_TYR250	−7.88	−2.16	−5.73
B_ASN252	−26.27	−1.61	−24.66	B_TRP261	−32.38	−5.14	−27.24	B_ASN252	−10.56	−1.10	−9.46
B_GLU253	4.96	−3.15	8.12	B_LYS263	8.51	−3.35	11.87	B_GLU253	−74.76	2.11	−76.87
B_VAL262	−6.69	−1.30	−5.39	B_TRP268	−11.42	−4.34	−7.08	B_TRP261	−14.40	−4.51	−9.89
B_LYS263	10.56	−2.86	13.42	B_TYR270	−12.64	−2.22	−10.41	B_VAL262	−13.13	−1.90	−11.23
B_TRP268	−23.58	−4.52	−19.06	B_ALA273	0.13	−0.67	0.80	B_LYS263	−7.77	−5.86	−1.92
B_SER286	−22.70	−0.97	−21.73	B_SER286	−30.97	−1.54	−29.43	B_TRP268	−8.30	−5.73	−2.58
B_ASP288	−123.58	2.66	−126.24	B_ALA287	−6.14	−2.22	−3.91	B_GLN285	−28.64	−1.66	−26.97
B_TYR293	−8.42	−2.83	−5.58	B_TYR293	−35.64	−2.16	−33.48	B_SER286	−11.89	−3.62	−8.28
								B_ALA287	−2.02	−4.15	2.14
								B_ASP288	−102.75	−1.73	−101.02
								B_TYR293	−36.34	−0.55	−35.80
BE	−273.51	−22.72	−250.79		−141.16	−26.60	−114.56		−446.49	−32.02	−414.48
Total IE	−600.65	−29.95	−570.70		−609.67	−32.19	−577.48		−653.61	−36.90	−616.70

**Table 4:** Contribution of the interactions energy in kcal/mol of the pneumolysin binding residues in the 3 Å from peptides.

**Residue**	**Interaction Energy (IE)**	**VDW**	**Electrostatic**	**Residue**	**Interaction Energy (IE)**	**VDW**	**Electrostatic**	**Residue**	**Interaction Energy (IE)**	**VDW**	**Electrostatic**
GLFDIVKKLVSDF-NH_2_				ILAWKWAWWAWRR-NH_2_				DM3: GLFDIWKWWRWRR- NH_2_			
A_VAL372	−8.80	−2.38	−6.42	A_ALA370	−20.04	−0.46	−19.58	A_ALA370	−19.09	−0.70	−18.39
A_ASP403	−32.90	−2.12	−30.78	A_TYR371	−10.11	−0.71	−9.40	A_TYR371	−14.36	−1.36	−13.00
A_CYS428	−5.92	−1.00	−4.93	A_VAL372	−13.59	−3.62	−9.97	A_VAL372	−5.41	−3.94	−1.48
A_GLY430	−0.65	−1.74	1.09	A_GLN374	−24.99	−2.17	−22.81	A_TYR376	−12.58	−2.31	−10.27
A_LEU431	−3.75	−1.71	−2.04	A_TYR376	−5.75	−2.37	−3.38	A_ASN400	−20.32	−2.52	−17.80
A_ALA432	−21.32	−1.44	−19.88	A_ASN400	−22.38	−3.20	−19.18	A_ASP403	−86.32	−3.38	−82.94
A_TRP435	−17.03	−9.21	−7.82	A_GLY401	−16.43	−1.21	−15.22	A_THR405	−6.01	−1.07	−4.94
A_TRP436	−15.47	−3.21	−12.25	A_ASP403	−103.64	−2.79	−100.85	A_CYS428	−10.44	−0.70	−9.73
				A_CYS428	−9.50	−0.80	−8.70	A_GLY430	−2.79	−1.01	−1.77
				A_GLY430	−9.23	−1.49	−7.75	A_ALA432	−22.08	−1.67	−20.41
				A_ALA432	−36.03	−1.61	−34.42	A_TRP435	−17.63	−9.53	−8.11
				A_TRP435	−34.61	−8.84	−25.78	A_TRP436	−11.94	−4.26	−7.68
				A_TRP436	−21.79	−5.06	−16.73				
BE	−105.85	−22.82	−83.03		−328.09	−34.32	−293.77		−228.97	−32.44	−196.53
Total IE	−347.93	−26.00	−321.92		−662.23	−42.08	−620.15		−414.15	−40.62	−373.53

**Table 5:** Contribution of the interactions energy in kcal/mol of the pspA binding residues in the 3 Å from peptides.

**Residue**	**Interaction Energy (IE)**	**VDW**	**Electrostatic**	**Residue**	**Interaction Energy (IE)**	**VDW**	**Electrostatic**	**Residue**	**Interaction Energy (IE)**	**VDW**	**Electrostatic**
GLFDIVKKLVSDF-NH2				ILAWKWAWWAWRR-NH2				DM3: GLFDIWKWWRWRR- NH2			
PSPA_TYR567	−24.44	−1.74	−22.70	PSPA_TYR567	−22.11	−1.27	−20.84	PSPA_TYR567	−24.53	−1.26	−23.27
PSPA_ASN569	−23.76	−3.15	−20.60	PSPA_ASN569	−14.12	−2.53	−11.60	PSPA_ASN569	−10.62	−2.80	−7.82
PSPA_ASN571	−28.35	−2.02	−26.34	PSPA_ASP573	−59.75	−2.12	−57.63	PSPA_MET574	−11.42	−0.09	−11.33
PSPA_ASP573	−22.37	−1.32	−21.06	PSPA_MET574	−11.51	−0.42	−11.09	PSPA_ALA575	−2.21	−2.57	0.36
PSPA_ALA575	−11.42	−3.10	−8.33	PSPA_ALA575	4.96	−2.73	7.69	PSPA_THR576	−23.29	−4.30	−18.99
PSPA_THR576	−11.42	−2.64	−8.78	PSPA_THR576	−48.20	−1.12	−47.09	PSPA_GLY577	−6.95	−1.25	−5.70
PSPA_TRP578	−14.02	−2.03	−11.99	PSPA_TRP578	−28.08	−4.24	−23.84	PSPA_TRP578	−28.77	−5.06	−23.71
PSPA_ALA579	−13.89	−2.55	−11.34	PSPA_ALA579	−24.12	−3.28	−20.84	PSPA_ALA579	−4.53	−1.45	−3.08
PSPA_LYS580	−3.99	−7.97	3.99	PSPA_LYS580	19.30	−5.09	24.38	PSPA_LYS580	−14.01	−3.91	−10.10
PSPA_VAL581	−8.50	−4.37	−4.13	PSPA_TRP585	−28.13	−5.71	−22.41	PSPA_VAL581	−14.75	−2.03	−12.72
PSPA_HIS582	−11.49	−0.90	−10.59	PSPA_GLU603	−89.33	−1.64	−87.69	PSPA_TRP585	−18.74	−4.85	−13.88
PSPA_TRP585	−33.49	−3.66	−29.82	PSPA_THR604	−21.75	−1.14	−20.61	PSPA_THR604	−17.50	−1.61	−15.90
				PSPA_TYR606	−16.79	−1.55	−15.24	PSPA_TYR606	−21.14	0.23	−21.37
				PSPA_LEU632	−17.09	−3.48	−13.62	PSPA_LEU632	−9.18	−3.18	−5.99
BE	−207.14	−35.45	−171.69		−356.73	−36.32	−320.41		−207.63	−34.12	−173.51
Total IE	−370.76	−39.45	−331.32		−758.96	−43.77	−715.19		−584.77	−40.77	−544.01
